# Technology in the management of type 2 diabetes – present status and future prospects

**DOI:** 10.1111/dom.14418

**Published:** 2021-05-20

**Authors:** Aideen Daly, Roman Hovorka

**Affiliations:** 1 Wellcome Trust-MRC Institute of Metabolic Science, University of Cambridge, Cambridge, United Kingdom

## Abstract

The growing incidence of type 2 diabetes (T2D) is a significant health concern, representing 90% of diabetes cases worldwide. As the disease progresses, resultant insulin deficiency and hyperglycaemia necessitates insulin therapy in many cases. It has been recognised that a significant number of people who have a clinical requirement for insulin therapy, alongside their healthcare professionals, are reluctant to intensify treatment with insulin due to fear of hypoglycaemia, poor understanding of treatment regimens or lack of engagement and are therefore at higher risk of developing complications from poor glycaemic control. Over the past decade, the rise of diabetes technologies, including dosing advisors, continuous glucose monitoring systems, insulin pumps and automated insulin delivery systems has led to great improvements in the therapies available particularly to those requiring insulin. Although the focus has largely been on delivering these therapies to the type 1 diabetes population, it is becoming increasingly recognised that people with T2D face similar challenges to achieve recommended glycaemic standards and also have the potential to benefit from these advances. In this review, we discuss diabetes technologies that are currently available for people with T2D and the evidence supporting their use, as well as future prospects. We conclude that there is a clinical need to extend the use of these technologies to the T2D population to curb the consequences of suboptimal disease management in this group.

## Introduction

Diabetes is a major global health problem affecting an estimated 463 million adults and a rising number of younger individuals worldwide. Approximately 90% of all cases of diabetes are type 2 (T2D) and the overall number is rapidly increasing^1^, causing significant burden for those affected and growing strain on healthcare resources. Despite the widening choice of oral and injectable anti-hyperglycaemic agents to treat T2D and the availability of long and fast-acting insulin analogs, many people struggle to achieve recommended glycaemic targets and are at risk of developing long-term micro- and macro-vascular complications^2^.

Although there is clear recognition of the need to intensify treatment for many people with T2D, timely and appropriate intensification of insulin therapy does not occur^3^, possibly due to fear of side-effects, individual choice or low health literacy. Recent advances in technology provide valuable means of improving the accuracy and safety of insulin therapy and simplifying glucose monitoring^4^. This should be harnessed to encourage appropriate intensification of treatment with the goal of improving outcomes.

To date, much research in diabetes technology has been directed towards treatment of type 1 diabetes (T1D)^5^ where a near absence of beta cell function necessitates lifelong insulin therapy. Hybrid closed-loop systems are now commercially available for people with T1D^6^ but evidence for its use in people with T2D lags behind. Although the pathophysiology of T2D differs, it is appropriate that the ability of technology to improve glycaemic control in T1D is extended to people with T2D.

This review discusses clinical evidence and diabetes technologies that have been applied in the treatment of T2D over the past decade. We explore technologies that are currently in use, possibilities for the future, and relevant guidelines from professional bodies on how these technologies can be utilised.

We have reviewed relevant literature available from Pubmed on diabetes technology in people with T2D from January 2010 until November 2020 using search terms: ‘closed-loop’, ‘hybrid closed-loop’, ‘artificial pancreas’, ‘self-monitoring blood glucose’, ‘continuous glucose monitor’, ‘flash glucose monitoring’, ‘type 2 diabetes’, ‘insulin pump therapy’, ‘continuous subcutaneous insulin infusion’ ‘diabetes technology’, ‘digital technology’, ‘mobile phone’ and ‘smart insulin pen’. Studies involving pregnant women are excluded, as are studies investigating non-technology pharmacological approaches.

## Contemporary diabetes technologies

**Figure 1:**
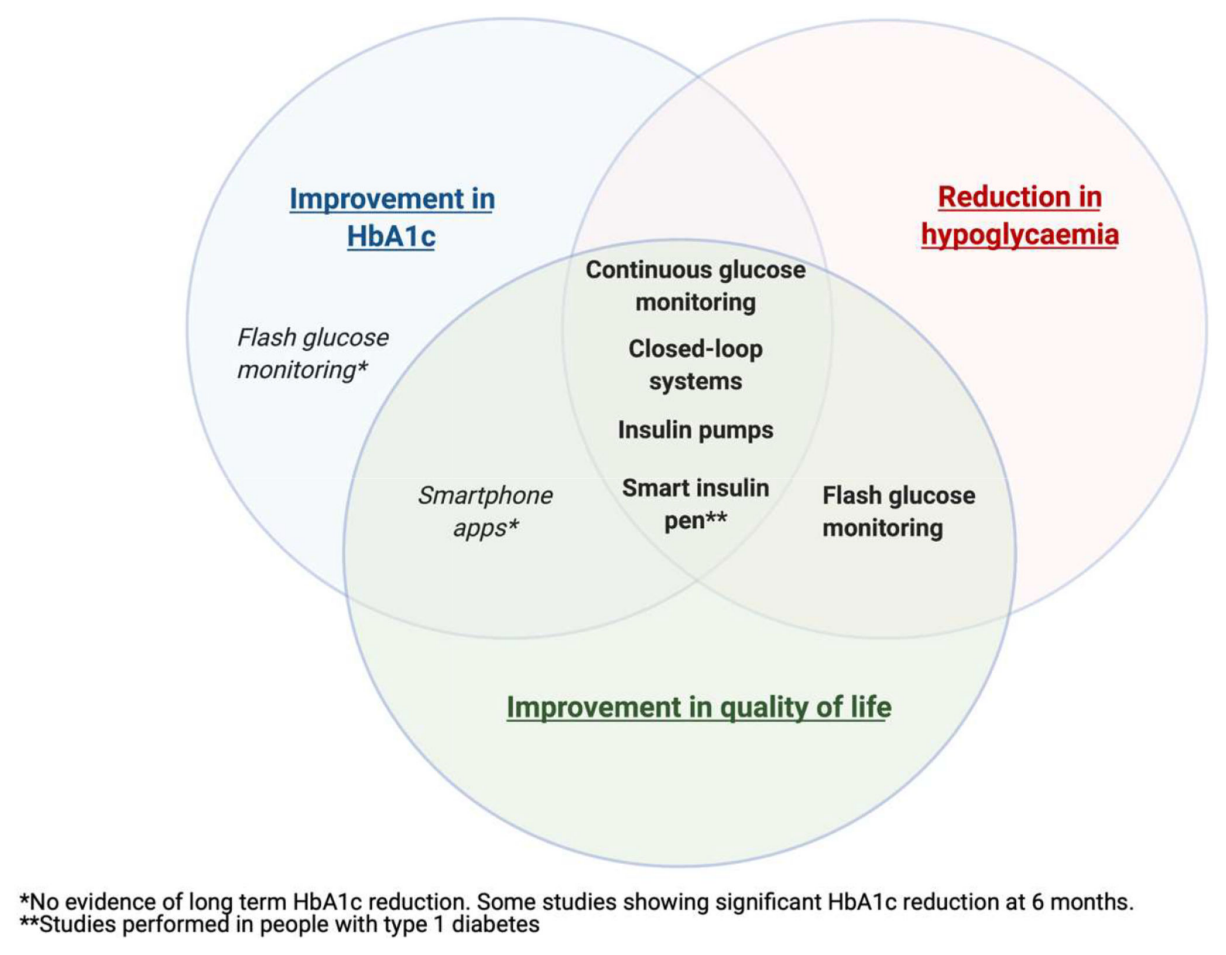
Clinical benefits of contemporary diabetes technologies Venn diagram illustrating contemporary diabetes technologies and associated clinical benefits based on review of available literature.

Figure 1 displays contemporary diabetes technologies available for use by people with T2D, and the associated clinical benefits.

Traditionally, T2D has been managed in a stepwise approach beginning with lifestyle interventions, progressing to one or more oral anti-hyperglycaemic agents, and ultimately many people require insulin therapy as the disease progresses^7, 8^. An estimation of global insulin requirements for type 2 diabetes between 2018-30 suggests that if insulin becomes widely available and is used appropriately to achieve HbA1c of less than 7%, approximately 15.5% of people with T2D worldwide will require insulin therapy^9^.

In the past decade, many advances in diabetes technology have focused on safer and more accurate glucose measurement and insulin delivery, therefore it should be acknowledged that these technologies will be of particular advantage to those on insulin therapy. The spectrum of new technologies spans from motivational smartphone apps which can benefit non-insulin requiring users, to modification of insulin pens, and simplified insulin pumps that integrate with continuous glucose monitors to function as an ‘artificial pancreas’ for those on insulin therapy. For older adults with high co-morbidity burden, these technologies may be less appropriate. A key focus should be the adoption of a user-centred approach, considering individual preference, technical ability and treatment goals to allow appropriate integration of incoming technologies into standard care.

## Technology enabled self-management

For some people with early T2D, evidence has shown reversibility of the disease with lifestyle modification^10^. For this reason, particular attention is given to promotion of exercise, healthy diet and education on minimising cardiovascular risk^8^.

Coinciding with an increase in smartphone use over the past decade, a number of smartphone applications (apps) have been designed to empower users with T2D to engage with lifestyle modification and promote diabetes self-management. Literature has been conflicting, with a number of small studies and randomised controlled trials (RCTs) reporting improvements in HbA1c, cardiovascular benefits and positive feedback from users and healthcare professionals following use of a smartphone app^11–16^. Other studies have been less encouraging, with an RCT by Agarwal *et al* (n=223) showing no HbA1c reduction and no improvement in quality of life or diabetes self-efficacy following use of an FDA-approved app ‘BlueStar’, possibly linked to a low app usage^17^. A qualitative study by Torbjørnsen *et al* also suggested low acceptability of apps and higher clinical and digital distress during periods of use^18^.

In line with the findings of a meta-analysis of 14 studies by Hou *et al* 19, it can be concluded that smartphone applications may be considered as an adjunct to improve HbA1c in people with T2D but acceptability and functionality must align with the digital literacy of the target population. For those unfamiliar with smartphone technology, a more practical approach may involve increased education and greater involvement of primary care to promote cardiovascular health^20^.

## Glucose monitoring

In contrast to the T1D population with a minimum recommended blood glucose measurement frequency of four times daily^21^, the requirement for glucose monitoring in people with T2D is less clear^8^. The National Institute for Health and Care Excellence (NICE) does not recommend SMBG for people with non-insulin treated T2D unless during pregnancy or in people at risk of hypoglycaemia, due to minimal clinical benefit, increased burden and cost^22, 23^. An RCT involving 450 people with non-insulin treated T2D showed no improvement in HbA1c or quality of life in those using SMBG compared to those who did not^24^.

### Self-monitoring of blood glucose (SMBG)

In people with insulin-treated T2D, literature is conflicting on the benefits of SMBG. An RCT by Nauck *et al* showed no improvement in glycaemic control with weekly SMBG profiling compared to no SMBG in people with T2D on conventional insulin therapy (basal insulin once daily or pre-mixed insulin twice daily). Treatment intensification was more likely in the monitoring group, suggesting earlier identification of the need for therapy adjustments ^25^. A meta-analysis involving 5,454 people with T2D in 24 studies concluded that glycaemic control was superior in SMBG groups compared to control groups at 12 and 24 weeks, with greater improvements seen in those with higher baseline HbA1c^26^. At 1 year, however, this did not reach significance, suggesting no sustained benefits of SMBG on glycaemic control. Of note, only 4 of the 24 studies included people on insulin therapy^26^.

Burden and costs associated with SMBG may reduce user’s engagement. Furthermore, ‘point-in-time’ glucose measurements and the absence of information on glucose trends or variability limit the clinical utility of SMBG and fails to detect nocturnal or asymptomatic hypoglycaemia. With the ability to overcome these limitations, flash and continuous glucose monitoring have gained popularity. These devices provide instantaneous information on glucose trends and variability, warnings for out-of-range glucose values as well as the ability to remotely review glucose profiles, allowing healthcare professionals and users to make required adjustments^27^.

### Flash glucose monitoring

A novel approach to glucose monitoring available since 2016 involves the use of a factory calibrated 14-day flash glucose monitor, FreeStyle Libre^28^ (Abbott Diabetes Care, CA, USA). A small circular sensor containing a thin fibre is inserted in the upper arm and records interstitial glucose every minute. 8 hours-worth of glucose values are stored by the device, therefore a minimum of 3 scans per day at 8-hour intervals enables recording of a full day of glucose values. Using a handheld reader or ‘near field communication’ enabled smartphone to scan the sensor results in production of an ambulatory glucose profile (AGP), providing a minimally invasive and rapid method of glucose monitoring and the ability to assess glucose trends and variability throughout the day and night^29^. Studies have shown high user satisfaction due to fewer finger pricks, ease of use and insertion of the device^30–33^.

A number of RCTs and a meta-analysis by Castellana *et al* (2020) showed improvements in HbA1c and reduced time in hypoglycaemia with the use of flash glucose monitoring compared to SMBG in insulin-treated T2D^33–35^. To the contrary, a multicentre RCT by Haak *et al* (n= 224) and an RCT by Furler *et al* (n=299) showed no difference in HbA1c but greater time in range and less time in hypoglycaemia ^36, 37^ suggesting particular benefit for people prone to hypoglycaemia. It should be noted, however, that secondary endpoint analysis of the RCT by Haak *et al* showed significant HbA1c reduction in participants <65 years, suggesting an inverse relationship of HbA1c with age. Possible reasons for this include lower engagement with sensor scanning, less interaction with AGPs or a reluctance to alter insulin doses among the older age groups. The absence of HbA1c reduction following the use of masked professional-mode flash glucose monitoring seen in the RCT by Furler *et al* suggests that direct user interaction and adjustments to therapy may be the driving force behind HbA1c reduction. Despite this, people with T2D using the masked ‘Libre Pro’ reported increased understanding of therapies and improved self-management of their diabetes due to interaction with AGPs alongside their clinical team^35^.

Measurement accuracy of a glucose sensor underpins its clinical utility and safety. Accuracy is commonly assessed using the mean absolute relative difference (MARD), with lower values indicating increased accuracy against reference glucose values^38^. A MARD of ≤10% has been hypothesised to facilitate non-adjunctive use of glucose sensors, while improvements to achieve MARD of <10% has not been shown to improve glycaemic outcomes^39^. FreeStyle Libre has been shown to be consistent and accurate over a 14-day period of use with a MARD of 11.4%^30, 40^.

Cost wise, flash glucose monitoring is comparable to SMBG 8.3 times daily in the UK, which is above the average testing frequency seen in people with T2D^36^. From a health service perspective, increased use of resources with SMBG compared to flash glucose monitoring based on observations from the REPLACE trial estimate total annual costs of flash glucose monitoring in the UK to be 13% lower than SMBG in people with T2D^41^. Thus, reductions in complications, reduced fingerstick testing and fewer hospital admissions may deem this a cost-effective option.

Limitations of the first-generation flash glucose monitoring system include the lack of alerts for hypo- or hyperglycaemia which has been addressed in the newer FreeStyle Libre 2, which displays an alert for out-of-range glucose values along with a prompt to check glucose. A smaller and thinner FreeStyle Libre 3 with full CGM capabilities is planned to become available in the EU at the same price as previous versions^42^.

### Professional guidelines on the use of flash glucose monitoring

A Medtech innovation briefing by NICE in 2017 on the use of FreeStyle Libre stated the device could be used to replace routine blood glucose monitoring in people with T2D on insulin therapy, but highlighted that people on fixed doses of insulin may derive less benefit. Reflecting results from clinical trials, it was also noted that FreeStyle Libre may be particularly beneficial to people with T2D experiencing frequent hypoglycaemia^43^. Despite this, the use of FreeStyle Libre is yet to be incorporated into NICE guidelines for the management of T2D in adults. American Diabetes Association (ADA) have updated guidelines on use of the device, stating that for insulin–treated people with T2D who are not meeting glycaemic targets, intermittently scanned continuous glucose monitors can be used to lower HbA1c and/or reduce hypoglycaemia^44^.

### Continuous glucose monitoring

The limitations of flash glucose monitoring can be overcome by the use of continuous glucose monitors (CGMs) which provide regular, real-time data and alerts/alarms without the need to physically scan a sensor. Improved design and accuracy has accelerated their use particularly among people with T1D, with more evidence emerging for their use in T2D.

Historically, HbA1c measurements and SMBG have been the primary methods to assess glycaemia in people with insulin-treated T2D, however there are limitations to these measures. HbA1c has been shown to be unreliable in certain groups^45^ and provides no information on glucose patterns, undetected hypoglycaemia or glucose variability. As a result, percentage of time in range captured by CGM systems is expected to supersede HbA1c as the preferred metric for assessing glycaemic control and risk of complications in T1D and T2D^27, 46^.

In contrast to the results of a number of RCTs showing no improvement in HbA1c with the use of flash glucose monitoring in T2D^36^, a RCT by Beck *et al* involving 158 adults with insulin-treated T2D using CGM technology over 24 weeks showed increased time in range and a reduction in HbA1c of at least 0.5% in 73% of the CGM group, compared to 49% of control group (adjusted difference in mean change in HbA1c from baseline -0.3% (CI -0.5% to 0.0%) p=0.022). This was consistent across all age groups, ranging from 35 to 79 years. Analysis of hypoglycaemia related endpoints was limited by the low percentage of time in biochemical hypoglycaemia and the absence of severe hypoglycaemia in both groups. Further studies with a follow-up period extending beyond 24 weeks would be beneficial in this regard, and to ensure HbA1c reductions are sustained.

Although quality of life measures did not differ significantly between CGM and control groups in this RCT, high user satisfaction was reported with CGM and more than 90% of users averaged 6 or more days of CGM use per week^47^.

A key benefit of CGM use in insulin-treated T2D is the ability to program alerts for hypoglycaemia. This is reflected in the International Consensus guidelines for the use of CGMs which advise that this technology ‘should be considered, along with HbA1c, to assess glycaemia in patients with insulin-treated T2D, especially if experiencing problematic hypoglycaemia’^27, 44^. Identification of asymptomatic and nocturnal hypoglycaemia on AGPs allow for earlier recognition of the need to dose adjust, and importantly, improves confidence that insulin therapy can be safely intensified.

Until recently, a deterrent to the adoption of CGMs in clinical practice was the need for calibration with fingerstick glucose to combat changes in sensor sensitivity over time. The release of a CGM with no requirement for calibration, Dexcom G6^48, 49^ (Dexcom, CA, USA), has enhanced quality of life for many users by eliminating this need to perform regular fingerstick testing.

Aside from the glycaemic benefits of CGM, it has been postulated that the ability to review continuous glucose data promotes healthy lifestyle practices and motivation to exercise^50, 51^, reducing insulin resistance and improving cardiovascular health in this high-risk group^52^.

### Implantable glucose monitors

The first implantable CGM (Eversense, Senseonics Inc, Germantown, MD, USA) has been available since 2016 and consists of a small cylindrical-shaped sensor inserted in the upper arm by a trained professional. Along with a wearable transmitter placed over the sensor and a smartphone application, this provides glucose data for up to 180 days 53. Three pivotal studies including patients with T2D were carried out to evaluate the safety and accuracy of the Eversense CGM and concluded that the system displayed accurate readings throughout the entirety of sensor life and had a favourable safety profile over traditional transcutaneous CGMs ^54–56^. The longer duration and implantable nature of the sensor makes this a preferable option for patients who dislike or have difficulty with frequent sensor changes, patients who may benefit from ‘on-body’ alerts via the vibrating mechanism of the transmitter, or patients with allergies to standard CGM adhesives ^53^. On the other hand, the inconvenience of a minor surgical procedure for insertion and removal of the device and the requirement to book a visit with a healthcare professional in the case of sensor failure is likely to deter some users ^57^. Currently, the 90-day implantable Eversense® CGM system (Senseonics, Senseonics Holdings, Inc, Maryland, USA) has been approved for non-adjunctive use in the USA, and the 180-day Eversense XL® system has been approved for adjunctive use in Europe.

Similar to flash glucose monitoring, ADA recommendations state that CGMs can be used to help lower HbA1c and/or reduce hypoglycaemia in adults with T2D who are not meeting glycaemic targets. In addition, the use of masked CGMs to characterise glycaemic patterns enabling earlier adjustments to therapy has also been highlighted as beneficial for people with T2D^44^. NICE guidelines for T2D do not currently include any recommendation for CGM use, however a NICE surveillance report in June 2019 flagged this as an area for review^58^. Table 1 shows currently available CGM systems for people with T2D.

## Insulin delivery

### Insulin pens

Insulin pens are the most widely used method of insulin administration in people with T2D^59^. The basic insulin pen delivers subcutaneous insulin from a cartridge via a disposable needle. Although this is a convenient approach for insulin administration, the requirement for manual record keeping of blood glucose readings, and the lack of connection to a digital ecosystem makes it challenging for healthcare professionals and users to interpret glucose profiles or assess dosing adherence.

Advances in insulin pen design over the last decade have resulted in the addition of memory function, caps, attachments, and ultimately ‘smart insulin pens’ with the ability to track doses and upload data to online platforms. ‘Memory’ function, whereby the insulin pen stores and displays information on previous bolus timing and amount, is particularly useful for people with cognitive impairment, or those with a lack of engagement in diabetes management due to the complexity of dosing regimens ^60^.

Bluetooth enabled insulin pen caps and attachments that link to smartphone apps allow users to track boluses, calculate remaining insulin, monitor insulin temperature and receive dosing reminders^61^.

Perhaps most useful is the ability of smart insulin pens to combine the above features with CGM data and upload to online platforms to enable healthcare professionals to remotely review and make adjustments to therapy^62, 63^. It has been shown that people with lower adherence to insulin dosing have poorer glycaemic control^64^, therefore smart pens may provide an effective method of highlighting those requiring education and support with behaviour modification at an earlier stage. Studies in the T1D population have shown increased dosing adherence and glycaemic improvements with smart pen use^65^. To our knowledge, no studies have been published on glycaemic outcomes, impact on quality of life or costeffectiveness of smart pen use in people with T2D. The ongoing clinical trial (NCT04678661) aims to investigate effects on glycaemic control, feasibility and usability of a smart pen consisting of a clip-on dose recorder with wireless connection to a ‘My Dose Coach smartphone app’ and provides dosing suggestions for people with T2D on once daily basal insulin. Table 2 presents currently available smart pens and associated features for use by people with T2D.

### Insulin pumps

Insulin pump therapy, or continuous subcutaneous insulin infusion (CSII), has been in use since the 1970’s and aims to mimic physiological insulin delivery for people with diabetes. Rapid-acting insulin is administered from a refillable reservoir into subcutaneous tissue at pre-programmed rates via a steel or plastic cannula which is replaced every 48-72 hours. Insulin pumps have become more user-friendly, compact and reliable resulting in increased popularity among people with diabetes, particularly in the paediatric T1D population^66^.

Current NICE guidelines advise against the use of CSII for people with T2D ^67^, and a consensus statement from ADA and European Association for the Study of Diabetes (EASD) in 2018 only briefly referred to a limited role for insulin pumps in a minority of people with T2D^23^. In recent years, evidence has been building that CSII in T2D is more effective and safer than conventional insulin therapy and indeed, the most recently updated ADA Standards of Medical Care in Diabetes (2021) recommend that insulin pumps can be considered as a treatment option for adults and youth with type 2 diabetes who are on MDI and able to manage the device 68.

Over the past decade, several small uncontrolled studies have shown improvements in glycaemic control, reductions in insulin requirements and improved quality of life for adults with T2D on CSII vs. multiple daily injections (MDI) or oral agents ^69–71^. These findings were corroborated by a retrospective observational study by Reznik *et al* involving 102 adults with T2D newly commenced on insulin pump therapy. 93% of participants were previously treated with insulin. HbA1c at 1 year improved from 9.3 ± 1.8% to 7.4 ± 1.4%, which was maintained at 6 years, suggesting long-lasting benefits of CSII use. Most notable HbA1c reductions were noted in those with baseline HbA1c level above 8% and those previously on oral diabetes drugs compared to basal-bolus therapy before initiation on insulin pump therapy^71^.

Larger RCTs including VIVID (n=365)^72^ OpT2mise (n=331)^73^ and a meta-analysis by Pickup *et al^66^* corroborated these findings of superior glucose control, lower total daily dose and high treatment satisfaction with CSII compared to MDI in adults with T2D^74^. Importantly, there was no increase in the incidence of hypoglycaemia^75^.

Pickup *et al* highlight an important concept of the above-mentioned studies, that the greatest HbA1c improvement was seen in the OpT2mise study which is unique in its inclusion of a 2-month run-in period prior to randomisation to optimise glycaemic control on basal bolus therapy. This infers that the cohort of people who struggle to achieve optimal glycaemic control after careful dose titration, dietary and physical interventions are most likely to benefit from CSII therapy.

Smaller studies comparing CSII to MDI use in T2D suggested a greater degree of weight gain with the former^71, 76, 77^, which is an important consideration given the higher cardiovascular risk in this group compared to those without diabetes. The results of the larger VIVID^72^ and OpT2mise^73^ RCTs, and meta-analysis by Pickup *et al^66^* were reassuring as no significant difference in weight gain was observed. As weight gain is a commonly reported reason for avoidance of insulin therapy in T2D, this is an important point to reiterate to clinicians and people who have expressed these concerns^78^.

Although many people with T2D have difficulty achieving glucose targets and therefore meet criteria for insulin therapy, a major barrier to treatment intensification is the fear of hypoglycaemia. It is known that severe hypoglycaemia in people with T2D is associated with microvascular and macrovascular adverse events and death^79, 80^. This has resulted in healthcare professionals inappropriately adopting ‘safer’ and often insufficient insulin intensification, so defined as ‘clinical inertia’. Although this applies to all methods of insulin delivery, it could be argued that greater knowledge of MDI therapy and more flexibility to prescribe ‘safer’ doses to avoid hypoglycaemia may lead to preference for MDI over pump therapy. Limited expertise of healthcare providers in the use of insulin pumps in general may represent a barrier to widespread adoption of pump use.

As with all technology, it is important to consider usability to ensure the target population will derive the intended benefits. An RCT by Chamberlian *et al* assessed user perceptions and usability of two insulin pumps using a usability scale in 28 participants including 12 adults with T2D previously on MDI^81^. The study concluded that user perceptions of usability significantly influences attitudes towards commencing insulin pump therapy, even in younger age groups^81^. Similarly, Reznik *et al* showed that participants who were completely autonomous using their insulin pump, or participants who involved a nurse to manage their pump had greater HbA1c improvements compared to those who were only technically autonomous^71^. This highlights a need for simpler designs and user-friendly devices to encourage uptake across all ages. In older age groups, other barriers to uptake may include cognitive decline, eyesight problems or lack of manual dexterity^82^. For this reason, insulin pump manufacturers have focused on improving designs to reduce training needs and potential for user error^83^.

Despite concerns regarding the ability of older individuals to manage an insulin pump, it is encouraging that although 38% of participants in the insulin pump therapy group of the OpT2mise trial had cognitive impairment, this did not impact on insulin pump efficacy or safety for these users^84^. Other studies have been carried out in people with T2D to investigate the use of simplified and more affordable insulin pumps with no requirement for pump programming or detailed education sessions, for example the ‘V-Go’ 24-hour disposable patch pump^85^ and ‘PAQ’ wearable 3-day CSII device with pre-defined basal insulin and on demand boluses^86^. Significant improvements in HbA1c were noted and patients reported a preference for the simplified CSII device over insulin injections. These findings suggest that adjunctive technologies including bolus calculator, temporary basal rates and carbohydrate ratio determination with meals are not necessary to improve glycaemic control in this group^87^, and that insulin pump therapy can be further simplified in T2D, with the goal of encouraging more people to avail of it.

Although typically T2D has been associated with older age groups compared to T1D, it is worth noting that the incidence of T2D is increasing across all age groups. The ADA have now recommended screening for T2D in children from age 10 or at onset of puberty in children who are obese with risk factors^88^. It is foreseeable that the growing cohort of younger persons with T2D will be adaptable to the use of technology, and more likely to reap the benefits of tighter glucose control over a longer time period.

Despite higher initial acquisition costs of insulin pump therapy compared to MDI, there have been numerous studies demonstrating long-term cost-effectiveness of CSII therapy in T2D. This is mainly driven by reductions in diabetes related complications and lower daily insulin requirements on CSII compared to MDI^89, 90^. A simulation study of the OpT2mise study estimated an 8.3% overall relative risk reduction for any cardiovascular event, and lifetime discounted savings of USD 66,883 over 40 years with the use of CSII compared to MDI^91^. Optimisation of insulin therapy using CSII has also been shown to reduce the need for concomitant oral anti-hyperglycaemic agents, reducing costs and polypharmacy^92^. For people with T2D and healthcare systems, fewer hospital admissions, emergency department attendances and clinic appointments provide a strong argument for the cost-effectiveness of this therapy

## Glucose responsive insulin delivery

The challenge of tailoring insulin therapy to manage glucose excursions in T2D can be overcome with the use of glucose responsive insulin delivery, commonly referred to as ‘closed-loop’ or ‘artificial pancreas’ systems. Real-time glucose measurements from a CGM device are communicated to a control algorithm which computes and directs insulin delivery via an insulin pump.

Earlier features of glucose responsive insulin delivery systems include the low glucose suspend feature which interrupts insulin delivery below a defined glucose threshold, and predictive low glucose suspend which predicts pending hypoglycaemia and suspends insulin delivery in advance^93^. No studies have been performed to assess the efficacy of these features in reducing frequency and severity of hypoglycaemia in T2D, studies are limited to the T1D population^94, 95^

With recognition that post-prandial glycaemic excursions are particularly challenging, the next phase of development was a ‘hybrid’ closed-loop system which requires meal announcements and initiation of a pump-delivered meal bolus by the user. The basal rate is automated for the remainder of the time between meals. Studies of inpatient and outpatient use of this system in people with T1D led to the introduction of the first hybrid closed-loop system in the USA in 2017 for use in T1D (670G pump, Medtronic, CA, USA)^96^. Since then a number of other hybrid closed-loop systems have been developed^6^.

The ultimate goal in reducing the burden of diabetes management is the fully automated closed-loop system which eliminates the need to undertake manual mealtime boluses. A number of RCTs have been carried out to assess the use of the fully closed-loop system for inpatients with T2D, and these have consistently shown increased time in target glucose range and no increased risk of hypoglycaemia, suggesting a safe and effective method of glucose control in this population^97–101^. Furthermore, the inclusion of inpatients on haemodialysis or artificial nutrition suggests that closed-loop insulin delivery is adaptable and effective in patients with co-morbidities complicating diabetes management^98, 101^. The largest RCT by Bally *et al* including 136 inpatients with T2D showed time in range of 65.8% with closed-loop therapy, compared to 41.5% in the control group. User satisfaction was high with 89% of users reporting that they were happy to have their glucose levels controlled by the system^97^.

To date, no studies have been published on the use of the fully closed-loop system in T2D in the outpatient setting, however there are three studies ongoing to clarify if this is a safe, viable and cost-effective option for this population outside the hospital environment (NCT04025775, NCT04701424, NCT04233229).

## Future directions

The majority of research into diabetes technology has been carried out in developed countries, however it should be noted that the majority of people with T2D live in low-and middle-income countries^102^. Many of these devices are unattainable by these groups due to cost, poor health literacy and educational deprivation. Indeed, in developed countries many people with type 1 and type 2 diabetes currently send fund diabetes technologies that are not routinely available from their healthcare provider^103^. A key challenge is ensuring equity of access to diabetes technologies across all nations and socioeconomic groups. There is a requirement for less complex technologies with provisions for cognitive and sensory impairment in order to expand the user base for these technologies and improve quality of life for those with limited technical ability or older people with T2D. Further clinical trials including this subgroup of people with T2D are required.

Studies to date have consistently shown positive outcomes with insulin pump use in people with T2D sub-optimally controlled on MDI, however professional guidelines do not currently support routine use in this group. The integration of insulin pumps with other diabetes technologies developed in the past decade has opened the gateway to methods of optimally controlling blood glucose and minimising user burden, including closed-loop insulin delivery. Limited access to insulin pumps hinders widespread adoption of these technologies into routine care. Further evidence from RCTs and health economics assessments are required to support their adoption on a larger scale.

Closed loop systems which enable glucose responsive insulin delivery are now commercially available for people with T1D. Further research is warranted to assess the efficacy and safety of this treatment in T2D.

## Conclusions

Glucose control for people with T2D remains challenging, particularly in those with a severe insulin deficit necessitating exogenous insulin delivery. There is a clinical need for safer and more user-friendly methods of insulin delivery. Advances in diabetes technologies over the past decade have provided cost-effective and valuable tools to safely commence insulin therapy and manage the disease effectively. Studies in inpatients with T2D have shown promising results for fully closed-loop insulin therapy. Further research is required to determine if this can be extended to the outpatient population with T2D.

## Figures and Tables

**Table 1:** Currently available continuous glucose monitoring systems for use in people with type 2 diabetes

CGM system	Manufacturer	Sensor duration (days)	Territories
Dexcom G6	Dexcom, CA, USA	10	USA and Europe
FreeStyle Libre 1	Abbott Diabetes Care, CA, USA	14	USA and Europe
FreeStyle Libre 2	Abbott Diabetes Care, CA, USA	14	USA and Europe
GlucoMen Day	Menarini Diagnostics, Italy	14	Europe
Eversense	Senseonics, MD, USA	90	USA
Eversense XL	Senseonics, MD, USA	180	Europe
S7 EasySense	Medtrum, China	14	Europe
Guardian Connect	Medtronic, MN, USA	6	USA and Europe

**Table 2:** Currently available smart pens for use in people with type 2 diabetes

Pen	Year of release	Manufacturer	Features	Bluetooth	Connection to online data repository/ app	Compatible insulin
NovoPen 5	2015	Novo Nordisk	- Displays time and dose of last injection. - Battery life and memory status ^104^	No	No	Penfill 3ml cartridges
NovoPen 6	2019	Novo Nordisk	- Tracks insulin doses ^102^ - Supported treatment decisions	No	Near Field Communication to compatible app (Diasend, mySugr) on Apple and Android devices	Novo Nordisk Tresiba or Fiasp cartridges
Esysta BT	2016	Emperra	- Displays time and dose of last injection - Countdown to next injection - Stores data on last 1000 injections - Temperature warning ^105^	Yes	Connects to Cloud-based ESYSTA portal and mobile app on Apple and Android devices.	Eli Lilly, Novo Nordisk and Sanofi Aventis 3ml cartridges
InPen	2016	Medtronic	- Tracks insulin on board - Dose reminder - Cartridge replacement reminder - Glucose check reminder - Temperature warning - Battery life status - Automatically logs doses^63^	Yes	- Connects to InPen app on Apple devices - Integrates with Bluetooth connected glucose meters and CGMs	Insulin aspart or lispro 3ml cartridges (Novolog, Humalog, Fiasp)
Pendiq 2.0	2017	Diamesco	- Displays remaining insulin - Alarms for double dosing - Records last 100 injections, dose and time ^106^	Yes	- Connects to Dialife app on Apple and Android devices. - Connects to Diasend platform via USB cable	Eli Lilly, Novo Nordisk, Sanofi-Aventis, Berlin- Chemie
YpsoMate SmartPilot	2017	YpsoMed	- Attaches to YpsoMate pen - Displays time and dose of last injection - Provides guidance throughout injection process - Dose reminder via app ^107^	Yes	Bluetooth transmission of injection events to mobile application	Prefilled insulin pens

## References

[1] International Diabetes Federation (2019). IDF Diabetes Atlas.

[2] Khunti K, Wolden ML, Thorsted BL, Andersen M, Davies MJ (2013). Clinical inertia in people with type 2 diabetes: a retrospective cohort study of more than 80,000 people. Diabetes Care.

[3] Reach G, Pechtner V, Gentilella R, Corcos A, Ceriello A (2017). Clinical inertia and its impact on treatment intensification in people with type 2 diabetes mellitus. Diabetes Metab.

[4] Cappon G, Vettoretti M, Sparacino G, Facchinetti A (2019). Continuous Glucose Monitoring Sensors for Diabetes Management: A Review of Technologies and Applications. Diabetes Metab J.

[5] Dovc K, Battelino T (2020). Evolution of Diabetes Technology. Endocrinol Metab Clin North Am.

[6] Leelarathna L, Choudhary P, Wilmot EG (2020). Hybrid closed-loop therapy: Where are we in 2021?. Diabetes Obes Metab.

[7] Ceriello A, deValk HW, Guerci B (2020). The burden of type 2 diabetes in Europe: Current and future aspects of insulin treatment from patient and healthcare spending perspectives. Diabetes Res Clin Pract.

[8] National Institute for Health and Care Excellence (NICE) NICE guideline (NG) 28 Type 2 diabetes in adults: management.

[9] Basu S, Yudkin JS, Kehlenbrink S (2019). Estimation of global insulin use for type 2 diabetes, 2018-30: a microsimulation analysis. Lancet Diabetes Endocrinol.

[10] Knowler WC, Barrett-Connor E, Fowler SE (2002). Reduction in the incidence of type 2 diabetes with lifestyle intervention or metformin. N Engl J Med.

[11] Quinn CC, Clough SS, Minor JM, Lender D, Okafor MC, Gruber-Baldini A (2008). WellDoc mobile diabetes management randomized controlled trial: change in clinical and behavioral outcomes and patient and physician satisfaction. Diabetes Technol Ther.

[12] Årsand E, Frøisland DH, Skrøvseth SO (2012). Mobile health applications to assist patients with diabetes: lessons learned and design implications. J Diabetes Sci Technol.

[13] Arsand E, Tatara N, Østengen G, Hartvigsen G (2010). Mobile phone-based self management tools for type 2 diabetes: the few touch application. J Diabetes Sci Technol.

[14] Whitehead L, Seaton P (2016). The Effectiveness of Self-Management Mobile Phone and Tablet Apps in Long-term Condition Management: A Systematic Review. J Med Internet Res.

[15] Frias J, Virdi N, Raja P, Kim Y, Savage G, Osterberg L (2017). Effectiveness of Digital Medicines to Improve Clinical Outcomes in Patients with Uncontrolled Hypertension and Type 2 Diabetes: Prospective, Open-Label, Cluster-Randomized Pilot Clinical Trial. J Med Internet Res.

[16] Kleinman NJ, Shah A, Shah S, Phatak S, Viswanathan V (2017). Improved Medication Adherence and Frequency of Blood Glucose Self-Testing Using an m-Health Platform Versus Usual Care in a Multisite Randomized Clinical Trial Among People with Type 2 Diabetes in India. Telemed J E Health.

[17] Agarwal P, Mukerji G, Desveaux L (2019). Mobile App for Improved SelfManagement of Type 2 Diabetes: Multicenter Pragmatic Randomized Controlled Trial. JMIR Mhealth Uhealth.

[18] Torbjørnsen A, Ribu L, Rønnevig M, Grøttland A, Helseth S (2019). Users’ acceptability of a mobile application for persons with type 2 diabetes: a qualitative study. BMC Health Serv Res.

[19] Hou C, Carter B, Hewitt J, Francisa T, Mayor S (2016). Do Mobile Phone Applications Improve Glycemic Control (HbA1c) in the Self-management of Diabetes? A Systematic Review, Meta-analysis, and GRADE of 14 Randomized Trials. Diabetes Care.

[20] Lean ME, Leslie WS, Barnes AC (2018). Primary care-led weight management for remission of type 2 diabetes (DiRECT): an open-label, cluster-randomised trial. Lancet.

[21] National Institute for Health and Clinical Excellence (NICE) NICE guideline (NG) 17, Type 1 diabetes in adults: diagnosis and management.

[22] National Institute for Health and Clinical Excellence (NICE) NICE guideline. Managing blood glucose in adults with type 2 diabetes.

[23] Davies MJ, D’Alessio DA, Fradkin J (2018). Management of hyperglycaemia in type 2 diabetes, 2018. A consensus report by the American Diabetes Association (ADA) and the European Association for the Study of Diabetes (EASD). Diabetologia.

[24] Young LA, Buse JB, Weaver MA (2017). Glucose Self-monitoring in Non-Insulin-Treated Patients With Type 2 Diabetes in Primary Care Settings: A Randomized Trial. JAMA Intern Med.

[25] Nauck MA, Haastert B, Trautner C, Müller UA, Heinemann L (2014). Diabetes-Gesellschaft) CTSGotGAftSoDD. A randomised, controlled trial of self-monitoring of blood glucose in patients with type 2 diabetes receiving conventional insulin treatment. Diabetologia.

[26] Machry RV, Rados DV, Gregório GR, Rodrigues TC (2018). Self-monitoring blood glucose improves glycemic control in type 2 diabetes without intensive treatment: A systematic review and meta-analysis. Diabetes Res Clin Pract.

[27] Danne T, Nimri R, Battelino T (2017). International Consensus on Use of Continuous Glucose Monitoring. Diabetes Care.

[28] Hoss U, Budiman ES (2017). Factory-Calibrated Continuous Glucose Sensors: The Science Behind the Technology. Diabetes Technol Ther.

[29] Abbott Diabetes Care FreeStyle Libre Flash Glucose Monitoring System.

[30] Bailey T, Bode BW, Christiansen MP, Klaff LJ, Alva S (2015). The Performance and Usability of a Factory-Calibrated Flash Glucose Monitoring System. Diabetes Technol Ther.

[31] Bolinder J, Antuna R, Geelhoed-Duijvestijn P, Kröger J, Weitgasser R (2016). Novel glucose-sensing technology and hypoglycaemia in type 1 diabetes: a multicentre, nonmasked, randomised controlled trial. Lancet.

[32] Edge J, Acerini C, Campbell F (2017). An alternative sensor-based method for glucose monitoring in children and young people with diabetes. Arch Dis Child.

[33] Yaron M, Roitman E, Aharon-Hananel G (2019). Effect of Flash Glucose Monitoring Technology on Glycemic Control and Treatment Satisfaction in Patients With Type 2 Diabetes. Diabetes Care.

[34] Castellana M, Parisi C, Di Molfetta S (2020). Efficacy and safety of flash glucose monitoring in patients with type 1 and type 2 diabetes: a systematic review and metaanalysis. BMJ Open Diabetes Res Care.

[35] Ajjan RA, Jackson N, Thomson SA (2019). Reduction in HbA1c using professional flash glucose monitoring in insulin-treated type 2 diabetes patients managed in primary and secondary care settings: A pilot, multicentre, randomised controlled trial. Diab Vasc Dis Res.

[36] Haak T, Hanaire H, Ajjan R, Hermanns N, Riveline JP, Rayman G (2017). Flash Glucose-Sensing Technology as a Replacement for Blood Glucose Monitoring for the Management of Insulin-Treated Type 2 Diabetes: a Multicenter, Open-Label Randomized Controlled Trial. Diabetes Ther.

[37] Furler J, O’Neal D, Speight J (2020). Use of professional-mode flash glucose monitoring, at 3-month intervals, in adults with type 2 diabetes in general practice (GP-OSMOTIC): a pragmatic, open-label, 12-month, randomised controlled trial. Lancet Diabetes Endocrinol.

[38] Heinemann L, Schoemaker M, Schmelzeisen-Redecker G (2020). Benefits and Limitations of MARD as a Performance Parameter for Continuous Glucose Monitoring in the Interstitial Space. J Diabetes Sci Technol.

[39] Kovatchev BP, Patek SD, Ortiz EA, Breton MD (2015). Assessing sensor accuracy for nonadjunct use of continuous glucose monitoring. Diabetes Technol Ther.

[40] Sato T, Oshima H, Nakata K (2019). Accuracy of flash glucose monitoring in insulin-treated patients with type 2 diabetes. J Diabetes Investig.

[41] Hellmund R, Weitgasser R, Blissett D (2018). Cost Calculation for a Flash Glucose Monitoring System for Adults with Type 2 Diabetes Mellitus Using Intensive Insulin -a UK Perspective. Eur Endocrinol.

[42] Abbott Newsroom FreeStyle Libre 3 Sets New Peak in Diabetes Care.

[43] National Institute for Health and Care Excellence (NICE) (2017). FreeStyle Libre for glucose monitoring : Medtech innovation briefing [MIB110].

[44] American Diabetes Association (2020). 7. Diabetes Technology:. Diabetes Care.

[45] Krhač M, Lovrenčić MV (2019). Update on biomarkers of glycemic control. World J Diabetes.

[46] Vigersky RA, McMahon C (2019). The Relationship of Hemoglobin A1C to Time-in-Range in Patients with Diabetes. Diabetes Technol Ther.

[47] Beck RW, Riddlesworth TD, Ruedy K (2017). Continuous Glucose Monitoring Versus Usual Care in Patients With Type 2 Diabetes Receiving Multiple Daily Insulin Injections: A Randomized Trial. Ann Intern Med.

[48] Acciaroli G, Vettoretti M, Facchinetti A, Sparacino G (2018). Toward Calibration-Free Continuous Glucose Monitoring Sensors: Bayesian Calibration Approach Applied to Next Generation Dexcom Technology. Diabetes Technol Ther.

[49] Wadwa RP, Laffel LM, Shah VN, Garg SK (2018). Accuracy of a Factory-Calibrated, Real Time Continuous Glucose Monitoring System During 10 Days of Use in Youth and Adults with Diabetes. Diabetes Technol Ther.

[50] Bailey KJ, Little JP, Jung ME (2016). Self-Monitoring Using Continuous Glucose Monitors with Real-Time Feedback Improves Exercise Adherence in Individuals with Impaired Blood Glucose: A Pilot Study. Diabetes Technol Ther.

[51] Yoo HJ, An HG, Park SY (2008). Use of a real time continuous glucose monitoring system as a motivational device for poorly controlled type 2 diabetes. Diabetes Res Clin Pract.

[52] Sampath Kumar A, Maiya AG, Shastry BA (2019). Exercise and insulin resistance in type 2 diabetes mellitus: A systematic review and meta-analysis. Ann Phys Rehabil Med.

[53] Deiss D, Szadkowska A, Gordon D (2019). Clinical Practice Recommendations on the Routine Use of Eversense, the First Long-Term Implantable Continuous Glucose Monitoring System. Diabetes Technol Ther.

[54] Christiansen MP, Klaff LJ, Brazg R (2018). A Prospective Multicenter Evaluation of the Accuracy of a Novel Implanted Continuous Glucose Sensor: PRECISE II. Diabetes Technol Ther.

[55] Christiansen MP, Klaff LJ, Bailey TS, Brazg R, Carlson G, Tweden KS (2019). A Prospective Multicenter Evaluation of the Accuracy and Safety of an Implanted Continuous Glucose Sensor: The PRECISION Study. Diabetes Technol Ther.

[56] Kropff J, Choudhary P, Neupane S (2017). Accuracy and Longevity of an Implantable Continuous Glucose Sensor in the PRECISE Study: A 180-Day, Prospective, Multicenter, Pivotal Trial. Diabetes Care.

[57] Petrie JR, Peters AL, Bergenstal RM, Holl RW, Fleming GA, Heinemann L (2017). Improving the Clinical Value and Utility of CGM Systems: Issues and Recommendations: A Joint Statement of the European Association for the Study of Diabetes and the American Diabetes Association Diabetes Technology Working Group. Diabetes Care.

[58] National Institute for Health and Care Excellence (NICE) (2019). NICE guideline NG17, NG18, NG19 and NG28.

[59] Klonoff DC, Kerr D (2018). Smart Pens Will Improve Insulin Therapy. J Diabetes Sci Technol.

[60] Peyrot M, Barnett AH, Meneghini LF, Schumm-Draeger PM (2012). Insulin adherence behaviours and barriers in the multinational Global Attitudes of Patients and Physicians in Insulin Therapy study. Diabet Med.

[61] Sangave NA, Aungst TD, Patel DK (2019). Smart Connected Insulin Pens, Caps, and Attachments: A Review of the Future of Diabetes Technology. Diabetes Spectr.

[62] Diasend (2020). List of devices that can be uploaded via Glooko Transmitter.

[63] Gildon BW (2018). InPen Smart Insulin Pen System: Product Review and User Experience. Diabetes Spectr.

[64] Munshi MN, Slyne C, Greenberg JM (2019). Nonadherence to Insulin Therapy Detected by Bluetooth-Enabled Pen Cap Is Associated With Poor Glycemic Control. Diabetes Care.

[65] Adolfsson P, Hartvig NV, Kaas A, Møller JB, Hellman J (2020). Increased Time in Range and Fewer Missed Bolus Injections After Introduction of a Smart Connected Insulin Pen. Diabetes Technol Ther.

[66] Pickup JC, Reznik Y, Sutton AJ (2017). Glycemic Control During Continuous Subcutaneous Insulin Infusion Versus Multiple Daily Insulin Injections in Type 2 Diabetes: Individual Patient Data Meta-analysis and Meta-regression of Randomized Controlled Trials. Diabetes Care.

[67] National Institute for Health and Care Excellence (NICE) Technology appraisal guidance (TA151). Continuous subcutaneous insulin infusion for the treatment of diabetes mellitus.

[68] American Diabetes Asociation (2021). 7. Diabetes Technology:. Diabetes Care.

[69] Frias JP, Bode BW, Bailey TS, Kipnes MS, Brunelle R, Edelman SV (2011). A 16-week open-label, multicenter pilot study assessing insulin pump therapy in patients with type 2 diabetes suboptimally controlled with multiple daily injections. J Diabetes Sci Technol.

[70] Gentry CK, Cross LB, Gross BN, McFarland MS, Bestermann WH (2011). Retrospective analysis and patient satisfaction assessment of insulin pump therapy in patients with type 2 diabetes. South Med J.

[71] Reznik Y, Morera J, Rod A (2010). Efficacy of continuous subcutaneous insulin infusion in type 2 diabetes mellitus: a survey on a cohort of 102 patients with prolonged follow-up. Diabetes Technol Ther.

[72] Grunberger G, Bhargava A, Ly T (2020). Human regular U-500 insulin via continuous subcutaneous insulin infusion versus multiple daily injections in adults with type 2 diabetes: The VIVID study. Diabetes Obes Metab.

[73] Conget I, Castaneda J, Petrovski G (2016). The Impact of Insulin Pump Therapy on Glycemic Profiles in Patients with Type 2 Diabetes: Data from the OpT2mise Study. Diabetes Technol Ther.

[74] Chlup R, Runzis S, Castaneda J, Lee SW, Nguyen X, Cohen O (2018). Complex Assessment of Metabolic Effectiveness of Insulin Pump Therapy in Patients with Type 2 Diabetes Beyond HbA1c Reduction. Diabetes Technol Ther.

[75] Reznik Y, Cohen O, Aronson R (2014). Insulin pump treatment compared with multiple daily injections for treatment of type 2 diabetes (OpT2mise): a randomised open-label controlled trial. Lancet.

[76] Edelman SV, Bode BW, Bailey TS (2010). Insulin pump therapy in patients with type 2 diabetes safely improved glycemic control using a simple insulin dosing regimen. Diabetes Technol Ther.

[77] Wolff-McDonagh P, Kaufmann J, Foreman S, Wisotsky S, Wisotsky JA, Wexler C (2010). Using insulin pump therapy in poorly controlled type 2 diabetes. Diabetes Educ 2010.

[78] Harris S, Abrahamson MJ, Ceriello A (2020). Clinical Considerations When Initiating and Titrating Insulin Degludec/Liraglutide (IDegLira) in People with Type 2 Diabetes. Drugs.

[79] Zoungas S, Patel A, Chalmers J (2010). Severe hypoglycemia and risks of vascular events and death. N Engl J Med.

[80] Goto A, Arah OA, Goto M, Terauchi Y, Noda M (2013). Severe hypoglycaemia and cardiovascular disease: systematic review and meta-analysis with bias analysis. BMJ.

[81] Chamberlain JJ, Gilgen E (2015). Do perceptions of insulin pump usability impact attitudes toward insulin pump therapy? A pilot study of individuals with type 1 and insulin-treated type 2 diabetes. J Diabetes Sci Technol.

[82] Stephens EA, Heffner J (2010). Evaluating older patients with diabetes for insulin pump therapy. Diabetes Technol Ther.

[83] Schaeffer NE, Parks LJ, Verhoef ET (2015). Usability and training differences between two personal insulin pumps. J Diabetes Sci Technol.

[84] Reznik Y, Joubert M (2015). The OPT2MISE Study -A Review of the Major Findings and Clinical Implications. Eur Endocrinol.

[85] Mora PF, Sutton DR, Gore A (2020). Efficacy, safety and cost-effectiveness comparison between U-100 human regular insulin and rapid acting insulin when delivered by V-Go wearable insulin delivery device in type 2 diabetes. BMJ Open Diabetes Res Care.

[86] Mader JK, Lilly LC, Aberer F (2018). Improved glycaemic control and treatment satisfaction with a simple wearable 3-day insulin delivery device among people with Type 2 diabetes. Diabet Med.

[87] Aronson R, Cohen O, Conget I (2014). OpT2mise: a randomized controlled trial to compare insulin pump therapy with multiple daily injections in the treatment of type 2 diabetes-research design and methods. Diabetes Technol Ther.

[88] Xu H, Verre MC (2018). Type 2 Diabetes Mellitus in Children. Am Fam Physician.

[89] Roze S, Duteil E, Smith-Palmer J (2016). Cost-effectiveness of continuous subcutaneous insulin infusion in people with type 2 diabetes in the Netherlands. J Med Econ.

[90] David G, Gill M, Gunnarsson C, Shafiroff J, Edelman S (2014). Switching from multiple daily injections to CSII pump therapy: insulin expenditures in type 2 diabetes. Am J Manag Care.

[91] Wahlqvist P, Warner J, Morlock R (2018). Cost-effectiveness of Simple Insulin Infusion Devices Compared to Multiple Daily Injections in Uncontrolled Type 2 Diabetics in the United States Based on a Simulation Model. J Health Econ Outcomes Res.

[92] Lynch PM, Riedel AA, Samant N (2010). Resource utilization with insulin pump therapy for type 2 diabetes mellitus. Am J Manag Care.

[93] Rodbard D (2017). Continuous Glucose Monitoring: A Review of Recent Studies Demonstrating Improved Glycemic Outcomes. Diabetes Technol Ther.

[94] Chen E, King F, Kohn MA, Spanakis EK, Breton M, Klonoff DC (2019). A Review of Predictive Low Glucose Suspend and Its Effectiveness in Preventing Nocturnal Hypoglycemia. Diabetes Technol Ther.

[95] Bergenstal RM, Klonoff DC, Garg SK (2013). Threshold-based insulin-pump interruption for reduction of hypoglycemia. N Engl J Med.

[96] (2016). Minimed 670G: a hybrid closed-loop insulin delivery system. Med Lett Drugs Ther.

[97] Bally L, Thabit H, Hartnell S (2018). Closed-Loop Insulin Delivery for Glycemic Control in Noncritical Care. N Engl J Med.

[98] Bally L, Gubler P, Thabit H (2019). Fully closed-loop insulin delivery improves glucose control of inpatients with type 2 diabetes receiving hemodialysis. Kidney Int.

[99] Thabit H, Hartnell S, Allen JM (2017). Closed-loop insulin delivery in inpatients with type 2 diabetes: a randomised, parallel-group trial. Lancet Diabetes Endocrinol.

[100] Kumareswaran K, Thabit H, Leelarathna L (2014). Feasibility of closed-loop insulin delivery in type 2 diabetes: a randomized controlled study. Diabetes Care.

[101] Boughton CK, Bally L, Martignoni F (2019). Fully closed-loop insulin delivery in inpatients receiving nutritional support: a two-centre, open-label, randomised controlled trial. Lancet Diabetes Endocrinol.

[102] Li A, Hussain S (2020). Diabetes technologies -what the general physician needs to know. Clin Med (Lond).

[103] Marso SP, Daniels GH, Brown-Frandsen K (2016). Liraglutide and Cardiovascular Outcomes in Type 2 Diabetes. N Engl JMed.

[104] Emperra Digital Diabetes Care (2017). ESYSTA Personal and fully automatic.

[105] Pendiq Intelligent Diabetes Care (2017). PENDIQ 2.0.

[106] YDS Delivery Systems (2020). SmartPilot-Transforming YpsoMate into a smart product system.

